# Experiences of patient education among people affected by cardiovascular disease: a qualitative study based on Andragogy model

**DOI:** 10.1186/s12913-023-09622-1

**Published:** 2023-06-29

**Authors:** Negin Niksadat, Mohtasham Ghaffari, Ali Ramezankhani, Sakineh Rakhshanderou, Ali Vasheghani Farahani, Reza Negarandeh

**Affiliations:** 1 Department of Public Health, Faculty of health, Tehran Medical Sciences, Islamic Azad University, Tehran, Iran; 2 Department of Health Education and Promotion, School of Public Health & Safety, Shahid Beheshti University of Medical Sciences, Tehran, Iran; 3 Cardiac Primary Prevention Research Center, Cardiovascular Disease Research Institute, Tehran University of Medical Sciences, Tehran, Iran; 4 Nursing and Midwifery Care Research Center, School of Nursing and Midwifery, Tehran University of Medical Sciences, Tehran, Iran

**Keywords:** Patient education, Cardiovascular diseases, Andragogy model, Qualitative research

## Abstract

**Background:**

Patient education is a key component of patient care, positively affecting health promotion and self-care ability. In this regard, an extensive body of research supports the use of the andragogy model in patient education. The study aimed to explore the experiences of people with cardiovascular disease in patient education.

**Methods:**

This qualitative study involved 30 adult patients with cardiovascular disease who were hospitalized or had a history of hospitalization. They were purposively recruited with maximum variation from two large hospitals in Tehran, Iran. Data were gathered by conducting semi-structured interviews. Data collection was done by conducting semi-structured interviews. Then, the data were analyzed using directed content analysis and a preliminary framework based on six constructs of the andragogy model.

**Results:**

Data analysis resulted in the development of 850 primary codes, which were reduced to 660 during data reduction. These codes were grouped into nineteen subcategories under the six primary constructs of the andragogy model, i.e., need-to-know, self-concept, prior experience, readiness for learning, orientation to learning, and motivation for learning. The most common problems in patient education were associated with self-concept, previous experience, and readiness for learning components.

**Conclusion:**

This study provides valuable information about the problems of patient education for adults with cardiovascular disease. Correction of the issues identified can improve care quality and patient outcomes.

## Background

Cardiovascular disease (CVD) refers to a group of cardiovascular disorders and is currently the first leading cause of death worldwide. Over three-quarters of CVD-associated deaths occur in countries with a low or moderate income [1]. World Health Organization (WHO) statistics indicate Iran had a CVD-associated mortality rate of 350 per 100,000 people [2]. In this light, effective prevention and management of this disease are among the foremost national healthcare priorities that can be achieved through effective approaches [3].

Cardiac rehabilitation is a comprehensive secondary prevention program designed to reduce CVD complications risk and improve patient outcomes. The American Heart Association and the Canadian Cardiovascular Society recognize patient education (PE) as a crucial indicator of quality cardiac rehabilitation because of its central role in cardiac rehabilitation and standard care guidelines [4, 5]. PE is defined as “the process by which healthcare providers and others impart information to patients that will alter their health behaviors or improve their health status” [6]. It is essential for patient care, self-care, and health promotion. As required by standards, PE must be conducted from the time of admission to the time of discharge [7]. People with CVD, for example, encounter numerous self-management challenges, particularly after discharge, such as maintaining a proper diet and physical activity and taking medications. Some studies have demonstrated that PE for people with CVD promotes self-management behaviors and heart health, improves patient satisfaction and health-related quality of life, lowers healthcare-related costs, and reduces hospital readmissions [8–12]. Contrarily, a lack of effective PE can result in a series of adverse outcomes, such as frequent requests for healthcare services due to the complications of the underlying condition, increased healthcare-related costs, and a higher risk of affliction by chronic conditions [7]. Pre-discharge PE prepares the patient for successful self-management and continuation of recovery at home. Although PE is a standard of care that improves patients’ knowledge and readiness for hospital discharge [13], some studies have reported the poor status of PE in Iran. Barriers associated with the healthcare system may include shorter hospital stays and decreased opportunities and time for educating patients [14–16].

Also, several factors affect healthcare providers’ capacity/willingness to provide effective PE. These barriers include adopting a paternalistic approach to PE, staffing ratios, healthcare providers’ limited counseling skills or teaching ability, and their inadequate knowledge of adult learning principles [9, 13, 14, 17].

PE can contribute to improved self-management of various diseases, including CVD. Evidence shows that PE that is planned based on adult learning principles and individualized patient-centered approaches is more effective [10, 13]. Andragogy has emerged as one of the dominant frameworks and perhaps the best-known theory of adult learning for the past 40 years [18]. It has been considered the science and art of helping adults learn in a way that believes the learning needs of adults are different from those of children. Andragogy as a learning-centric framework consists of six principles that contrast with pedagogy, a teacher-centered approach [7, 19, 20]. Six main principles of andragogy include (1) adults need to know why they need to know something (need to know), (2) move toward greater self-directedness in learning (self-concept), (3) use the prior experience as a rich source of learning (prior experience), (4) need to be ready for learning (readiness for learning), (5) have a problem-centered approach to learning that is based on immediate application of learning in real life (orientation to learning), and (6) are motivated for learning more by internal factors than external factors (motivation for learning) [7, 20].

Applying these principles to PE provides PE experiences that impact individual, institutional, and societal growth. Accordingly, this approach may impact individual goals through improved health outcomes and personal empowerment for self-management, institutional goals that include increased patient satisfaction rates and improved patient care, and societal growth that will be evident through health promotion and knowledge in the local community [7, 18, 20].

Employing andragogy in PE enables healthcare providers to gain a better understanding of their clients’ educational needs and develop individualized patient-centered education [7, 20] that is crucial for preventing future problems and disease recurrences, especially in people with CVD [11]. Review studies revealed that regular use of andragogy positively affects patients’ adherence to PE [10, 21]. For example, people with CVD are motivated to adopt a healthier lifestyle when they perceive PE as useful and applicable in reducing heart disease risk [11]. Research also indicates that developing educational programs for patients based on adult learners’ characteristics can improve healthcare quality and provider satisfaction [10, 21]. On the other hand, since most people with CVD are adults, using andragogy principles can promote PE practice in this population. However, little is known about the experience of people with PE during their hospital stay. Consequently, this study aimed to explore the experiences of people with CVD in PE using the andragogy model.

## Methods

### Study design

This qualitative study was conducted using a directed content analysis approach to explore the experiences of PE among patients with CVD based on the principles of the andragogy model (a widely supported adult education model in PE).

The study also adheres to the Consolidated Criteria for Reporting Qualitative Research (COREQ) guidelines [22].

### Setting and participants

Participants were adults with CVD who were referred to Modares Hospital and Tehran Heart Center, Tehran, Iran, to receive inpatient or outpatient care services. They were purposively recruited to the study if they were hospitalized or had a history of hospitalization, had received PE, and agreed to share their PE experiences. Potential participants have approached face-to-face interviews focusing on explaining the study’s aim. Purposive sampling was done with maximum variation regarding participants’ age, gender, marital status, and educational level. Data collection and analysis were carried out concurrently, and sampling was completed with 30 patients when data saturation was reached.

### Data collection

Data were collected through semi-structured face-to-face interviews from October 2020 to May 2021. Interviews were guided using a broad, open-ended question, i.e., “How was the education you received about your illness?” Then, more specific questions were asked based on the principles of andragogy. Examples of these questions are, “Can you explain the understandability of the provided educational materials?” and “Can you express your opinion about the usefulness of the education?” Moreover, participants were encouraged to provide more detailed information about their experiences through probing questions such as, “Can you provide an example?” The duration of interviews varied from fifteen to thirty minutes, depending on the participants’ conditions. The first author (NN), a female Ph.D. candidate in Health Education and Promotion with extensive qualitative research expertise, conducted interviews in patient rooms for hospitalized patients or in a room in outpatient clinics affiliated with these hospitals. All interviews were recorded with participants’ consent and transcribed verbatim. Interviews continued until data saturation was reached and no new themes or ideas emerged. The outline of the interview is as follows (Table 1).

**Table 1:** Guideline of the interview

Categories	Specific questions
Broad, open-ended question	● I would like you to tell me about the education you received about your illness in the hospital. ● How was the education, in your opinion?
Need-to-know	●Would you please let me know your opinion on the usefulness of the topics you received?
Learners’ self-concept	● What role did you play in this education? In which part did you participate? (For example, what role did you play in deciding to choose the time, place, educational materials, or method of education?)
	● How could you ask your questions during education?
Learners’ prior experience	● Before starting the education, what aspects and characteristics of yourself or your illness were you asked about? What else did they ask about you?
	● Would you please explain the understandability of the provided educational materials?
Readiness for learning	● Would you please explain your conditions and readiness to receive educations?
	● How were the space, time, and place of education?
Orientation to learning	● How do you think these education sessions will help you, given your problem?
	● How does it make your tasks easier?
Motivation for learning	● In these education sessions, what motivated you the most to apply or learn them? What made you take them seriously?
Additional questions	● How do you think education will be improved? If you wanted to teach yourself, how would you teach?
	● Is there anything else you want to say?
	● Do you have any suggestions?

### Analysis

Data were analyzed through the directed content analysis method recommended by Shannon and Hsieh. With a directed approach, the analysis starts with a theory or relevant research findings as guidance for the initial code [23].

Accordingly, three experts in qualitative research carefully read each interview transcript several times to obtain an overall understanding. Then, the data were broken into meaning units and coded. Afterward, the codes were compared sequentially based on their similarity and difference (initial coding was compared, reviewed, and refined). These codes were categorized into six major andragogy components: need-to-know, self-concept, prior experience, readiness for learning, orientation to learning, and motivation for learning. Then, codes were compared, reviewed, discussed, and classified into subcategories according to their similarities and were labeled. We managed data using Microsoft Office (Word, Excel). MAXQDA software (v. 10.0) was also used for data management.

### Trustworthiness

Lincoln and Guba’s four criteria of credibility, transferability, confirmability, and dependability were utilized to ensure study rigor [24]. Credibility was established through prolonged engagement with participants in the field for about seven months and performing member and peer checking. This way, all interview texts, initial codes, and subcategories were reviewed and analyzed independently by three authors. The research team checked for agreement and discussed discrepancies to create a final coded data set. During member checking, participants were asked to verify the consistency of the textual material with their experiences. Transferability was established by providing detailed data about participants’ characteristics and the study context. Moreover, the age, gender, social and education levels of the patients, the selection process were considered, and the data collection and analysis were described as clearly as possible. The entire study process was meticulously documented to provide data confirmability. Ultimately, peers assessed the dependability of transcripts to ensure consistent decision making.

### Ethical considerations

The protocol of this study was approved by the Ethics Committee of Shahid Beheshti University of Medical Sciences, Tehran, Iran (code: IR.SBMU.PHNS.REC.1395.34). The procedures used in this study adhere to the tenets of the Declaration of Helsinki. Necessary permissions for the study were obtained from the university and provided to the authorities of the study setting. Also, informed consent was obtained from each participant before the actual data collection. The aim of the study was explained to the participants, and they ensured the confidentiality of their data and their freedom to withdraw from the study voluntarily. There was no prior relationship between the interviewer and the participants.

## Results

Thirty people with CVD participated in this study. Their mean age was 55.69 ± 13.01 years. Around 54% had coronary artery disease, 46% had high school diplomas, and 66% were housewives or retired (Table 2).

**Table 2:** Demographic items: counts and percentages

Variable	Frequency & Percent
Gender	
Female	18 (60%)
Male	12 (40%)
Age	
<30	3(10%)
30–50	9 (30%)
>50	15 (50%)
Education level	
primary school	7 (23.3%)
Less than high school	2 (6.6%)
Diploma	14 (46.6%)
Bachelor	6 (20%)
Master	1 (3.3%)
Job	
Unemployed	3 (10%)
Self-employment	5 (16.6%)
Employee	2 (6.6%)
Retired	8 (26.6%)
Housekeeper	12 (40%)
CVD disease	
Coronary artery	16 (53.3%)
Rheumatoid arthritis	3 (10%)
Congenital heart disease	2 (6.6%)
Cardiac arrhythmia	2 (6.6%)
Other cases	7 (23.3%)

During data analysis, 850 codes were identified, which were reduced to 660 during data reduction. These 660 codes were categorized into nineteen subcategories under the six main principles of andragogy. These categories and subcategories are explained in the following (Table 3).

**Table 3:** Categories and subcategories

categories (Principles of andragogy model)	subcategories
Need-to-know	● Usefulness and essentiality of education
	● PE as a complement to treatment
	● The necessity to inform patients about the usefulness of education
Learners’ self-concept	● Involvement of patients and attention and responsiveness to their questions
	● Self-directedness (assigning learning responsibility to patients)
Learners’ prior experience	● Selecting teaching method based on learners’ conditions and preferences
	● Congruence of education with patients’ educational level, experiences, knowledge, and understanding
	● Considering patients’ differences in PE (cultural differences, type of disease, physical condition, and learning ability)
Readiness for learning	● Considering patients’ physical and mental readiness and preferences
	● Appropriateness of the time and place for education
	● Getting patients ready for education
Orientation to learning	● Applicability of education
	● Practicability of education
Motivation for learning	● Adherence to education in order to protect and promote health
	● Feeling responsibility for self and family
	● Trust in education providers
	● Acquiring and imparting information
	● Personal characteristics
	● Understanding the applicability of education

**Need-to-know**: Adults need to know why they need to learn; **Learners’ self-conception**: adults want autonomy over what to learn, and they are self-directed learners; **Learners’ prior experience**: adults have experiences that can be a valuable resource in the process of learning; **Readiness for learning**: adults should be ready to learn; **Orientation to learning**: Adults’ orientation to learning is problem-centered; **Motivation for learning**: adults are primarily motivated to learn through internal motivations

### Need-to-know

The first main category of the study was the need-to-know. This category refers to the fact that adults need to know why they need to learn about that before learning or doing something. Moreover, they want to know how the learning experience will benefit them. This category was divided into three subcategories.

#### Usefulness and essentiality of education

Most participants felt the need for the provided educational materials. In their view, educational materials helped prevent disease recurrence and improve their knowledge, nutrition, and health.

*Education is a real need, particularly for older people, because they have limited health-related information and are primarily illiterate (a 66-year-old woman).*

#### PE as a complement to treatment

Participants considered PE as a complement to treatment. They noted that the lack of proper PE might result in disease recurrence and waste medical efforts.

*A woman who underwent coronary angiography was not provided education about physical activity permissible levels. She had ascended and descended stairs and done heavy tasks and hence, experienced bleeding and was hospitalized for nine days for bleeding and infection. You see that education is very important (a 49-year-old woman).*

#### The necessity to inform patients about the usefulness of education

Some participants pointed out healthcare providers do not adequately inform patients about PE’s benefits; hence, some consider education useless.

*Have you ever seen students’ happiness when their teacher skips class? Similarly, some patients thought they should miss educational sessions. It had not been justified to them that the educational session was intended for their benefit (a 75-year-old woman).*

### Learners’ prior experience

According to the principles of andragogy, learners’ prior experience is one of the essential key factors in adult learning. Our participants had different backgrounds and viewpoints due to their varied conditions.

#### Selecting teaching method based on learners’ conditions and preferences

Some participants needed and preferred receiving visual and written educational materials and face-to-face education. They noted that patients and their family members could use written educational materials at home. Accordingly, family members can get involved in providing education at home to their patients, mainly illiterate ones.

*After face-to-face education, illiterate patients should also receive an educational booklet that their family members can read to them at home. A one-size-fits-all approach cannot be used for all patients (a 49-year-old woman).*

Moreover, participants highlighted the importance of individualized education for each patient. Some noted that each patient might have unique questions depending on their underlying illness; thus, individualized education can allow him/her to address any possible questions.

*Even if doctors have limited time, I think individualized education, particularly at hospital discharge time, is better (a 70-year-old woman).*

#### Congruence of education with patients’ educational level, experiences, knowledge, and understanding

According to the participants, patients’ educational level, prior knowledge, and experiences were barely evaluated before PE.

*At school, there are thirty students at one level, for example, at the tenth- or twelfth-year level. But, patients are very diverse here. We have old, young, and illiterate patients who differ from each other in their underlying conditions (a 75-year-old woman).*

#### Considering patients’ differences in PE (cultural differences, type of disease, physical condition, and learning ability)

Patients are also different from each other, respecting their cultural background, underlying conditions, and physical and mental health status. A patient with a hearing problem who was satisfied with her PE experience expressed,

*The two doctors who came here to examine me noticed my hearing problem and gave me an understandable education (a 65-year-old woman).*

### Learners’ self-concept

According to the self-concept principle of andragogy, adult learners perceive themselves as responsible for their decisions and value the right to choose in learning. This main category contains two subcategories.

#### Involvement of patients and attention and responsiveness to their questions

Participants considered information provision to patients, responsiveness to their inquiries, and involvement in decision-making as their essential needs. They noted that fulfilling these requirements reduces stress for patients and their families while promoting self-confidence and tranquility. Nonetheless, they highlighted that some physicians refrained from informing patients and their family members or involving them in the treatment process.

*When we ask a question, the physician says, “There is no problem. We know our job. Don’t interfere”. They left me in that state of concern and uncertainty. After six years, a physician finally explained to me my heart problem, and thereby I found great calmness. Thank God (a 48-year-old woman).*

*I just listened and said “Ok” and had no more roles in education (a 41-year-old woman).*

#### Self-directedness (assigning the responsibility of learning to patients)

Although patients in one hospital were provided with a short brochure, if any, authorities in the other hospital provided them with educational booklets and thereby assigned the responsibility of learning to patients and their family members. Of course, none of the participating patients received educational CDs or online educational materials.

*Education* sessions *in the form of general recommendations about nutrition, physical exercise, and medications were good. But educational materials are not provided to patients using educational CDs to be used at home to help them take responsibility for self-management (a 58-year-old man).*

### Motivation for learning

In andragogy, the motivation for learning principle holds that adults are motivated to learn more through internal factors (such as quality of life) than external factors (such as higher income). The subcategories under this main category were adherence to education to protect and promote health, feeling responsibility towards self and family, trust in education providers, acquiring and imparting information, personal characteristics, and understanding the applicability of education. Findings showed that most patients were highly motivated to adhere to education, while some lacked adequate motivation.

*A reason for taking education seriously is that I don’t like disease recurrence and rehospitalization. Health is a blessing. I am a sensitive person and attempt to follow the education (a 49-year-old woman).*

*I need to adhere to the education to avoid getting myself, my family members, my children, and these nurses into trouble again (a 60-year-old woman).*

### Readiness for learning

Paying attention to learners’ learning readiness and preferences is one of the principles of andragogy. This main category had three subcategories.

#### Considering patients’ physical and mental readiness and preferences

Because of their different conditions, participants had different experiences and attitudes about the importance of readiness for learning. Some participants highlighted the importance of having physical and mental readiness, adequate calmness, and no stress during education. Therefore, some complained about education providers’ inattention to their readiness and physical and mental conditions during PE.

*I was not ready for education. I was in such severe pain that I didn’t understand anything (a 60-year-old man).*

#### Appropriateness of the time and place for education

Some participants noted that education sessions were provided to patients at inappropriate times, e.g., during rest, at hospital discharge, or in noisy environments. Moreover, they highlighted that the environment for group education was not proportionate to the number of patients.

*They provided education at their preferred time or whenever they had no other task. For instance, I was asleep when one of them suddenly came and asked me about my hand problem and provided education about the permitted level of hand activity (a 58-year-old man).*

*In this well-equipped hospital, at least one assembly hall on each floor for PE should ensure patient calmness during education. However, they provided education while I was in bed, and the two other patients in the room sat next to me. Anyway, although your colleague’s speech was very good and clear, the environment was not appropriate for education (a 48-year-old woman).*

#### Getting patients ready for education

Some participants noted that they had been prepared for education, while others had not previously been informed about group education sessions.

*They just came and selected some of us and said we should receive an education. We had not been informed and were not ready for education at all. Adequate readiness could help us ask our questions and better use education (a 58-year-old man).*

### Orientation to learning

The orientation to adult learning is problem-based and is based on the immediate application of education in real life. The two subcategories of this main category were applicability of education and practicability of education. Some education may be applicable but not practicable due to patients’ conditions, financial status, educational level, or family problems.

*We adhered to education according to our financial status. My spouse’s income is not very high (a 50-year-old woman).*

## Discussion

This study was the first qualitative study to explore the experiences of PE among people with CVD based on the andragogy model. Findings revealed that the need-to-know principle of andragogy was considered in education sessions. Participants understood the essentiality of PE and considered educational materials essential and useful. In line with these findings, in an andragogy-based study to develop and evaluate an educational program on CVD risk assessment, the learning needs of individuals in the educational process were considered and emphasized [25]. However, in Stefanie et al.‘s study into Patients’ Experiences of cardiovascular health education, patients reported unmet health information needs [11].

Study findings also indicated that participants had not been informed about the usefulness of education. The need-to-know principle of andragogy holds that before learning or doing something, adults need to know why they need to know and how the learning experience will benefit them [7, 20]. Also, effective PE based on adult learning principles begins with a holistic and patient-centered assessment of learning needs [10]. Health professionals should consider these points when planning PE in the future. However, a study reported that education providers did not inform patients about the reasons for learning educational materials because they assumed patients already knew the essentiality of education [26].

Respecting the orientation principle of andragogy, we found that most participants considered education applicable for complication prevention, disease recurrence, and health protection. Similarly, most former study participants highlighted the relevance of education to their problems [26]. Moreover, the education provided to patients with prostate cancer in another study was relevant to their daily problems and applicable to managing their disease [27]. According to andragogy principles, adults have a problem-based orientation to learning, meaning that education should be immediately applicable. In other words, when patients find learning useful for managing their diseases and daily lives, they will have greater motivation for learning [7, 10, 12, 20, 28]. These findings demonstrate the significance of focusing future PE programs on what adult patients need, find useful, and can apply in their daily lives to manage their health problems [10, 26].

Our findings also showed that healthcare providers disregarded patients’ readiness for learning. Previous studies have reported that such inattention is primarily due to the high number of patients, inadequate staff for education, physicians’ and nurses’ time limitation, and lack of adequate planning for education [7, 13, 16]. In a previous study, patients suggested that the delivery of education while they were tired or resting and the provision of extensive educational materials at the time of hospital discharge were among the main problems of PE [7]. This finding is consistent with our study. Interestingly, in the study by Sanchez et al. on using adult learning principles to facilitate PE, patients were allowed to review the information when they were ready and at their own pace [9].

All these findings denote that nurses should gain a deeper understanding of learning barriers such as pain and anxiety. They should also consider patients’ varying preferences and conditions to set the time and place of learning to create an atmosphere of trust and respect [9, 11, 29].

Findings revealed that most patients had adequate motivation for adherence to education. Their internal motives for learning included their responsibility towards their families, their accurate understanding of the applicability of education, and their positive attitudes toward the effectiveness of education in protecting and promoting their health, improving their self-management ability, and reducing their dependence on others. Of course, some participants had limited motivation for learning due to factors such as obstinacy, heavy workload, despair, stress, depression, and education providers’ inappropriate conduct. According to the andragogy model, adherence to education because of its perceived applicability confirms the assumption that motivation for learning is influenced by the individual’s perception of the usefulness, essentiality, and applicability of education [20]. According to the andragogy model, adherence to education because of its perceived applicability confirms the assumption that motivation for learning is influenced by the individual’s perception of the usefulness, essentiality, and applicability of education [11].

Different studies highlighted the importance of strengthening learners’ motivation and encouraging them to learn through education providers [11, 30–32]. Therefore, it is recommended that healthcare providers pay more attention to creating internal motivation in patients, primarily by providing applicable education.

Our findings also indicated that information provision to patients and their involvement in the treatment process were among their essential needs. Such information provision and involvement can reduce patients’ stress and uncertainty and promote their calmness. Allocating adequate time to talk with patients, providing them with information, and answering their questions develop their trust in healthcare providers, promoting their adherence to education and improving treatment outcomes. Moreover, patients’ active physical and mental involvement in learning through question and answering improves learning effectiveness [10, 11, 20]. Consequently, these considerations and the significance of patient involvement in planning future PE programs should be taken into account by health professionals [10, 11, 33]. In line with these findings, another study based on adult learning principles found self-directed learning and creating an atmosphere of trust and respect by addressing patients’ questions and concerns as one of the essential aspects of PE [9].

We also observed that while patients in one of the hospitals were not provided with significant written educational materials, authorities at another hospital provided educational booklets to patients to promote their self-directed learning. Patients in an earlier study were also interested in educational materials which could be used at home [10]. Providing patients with educational resources, including educational sites they can use after returning home, is highly recommended [21]. There are different reasons for the non-involvement of patients in education and disregarding the self-concept principle of andragogy. These reasons include education providers’ presumptions about patients’ incompetence in decision-making, their paternalistic approach to patient management, their time limitation, and the high number of patients [26].

Patients differ from each other, respecting characteristics such as contexts, learning styles, motivation, needs, efforts, beliefs, and goals. These differences should be considered in education [20]. Our participants’ educational needs and preferences varied according to their medical conditions. For instance, some preferred visual and written educational materials and face-to-face education. On the other hand, some also noted the potential of written materials in encouraging family members to get involved in PE, particularly for illiterate patients. In line with these findings, a former study found that patients had different preferences regarding educational resources and approaches [10]. Thus, using different educational approaches based on patients’ preferred learning styles can significantly improve PE effectiveness [9–11].

Our participants even preferred individualized education at the time of hospital discharge. Similarly, another study stressed the importance of patient-centered education based on each CVD patient’s unique needs and learning style [11]. This preference aligns with the prior experience principle of andragogy, which suggests individualized education [20]. Nonetheless, we identified several problems in PE, such as providing similar education sessions to all patients irrespective of their educational levels and knowledge background and providing simple and repetitive educational materials as general recommendations. Similarly, a previous study of patients with breast cancer demonstrated a lack of patient assessment before education, resulting in patients receiving an excessive amount of inappropriate information during education [34]. Patient assessment before education can turn education into a more positive experience and enhance patient satisfaction [9, 10, 29, 35]. Many factors contribute to education providers’ inattention to the prior experience principle of andragogy, including time limitations, staff shortage, and a high patient load.

Healthcare providers must pay attention to the differences between patients, avoid providing uniform and routine education to all patients without leveling them and pay attention to their differences concerning education level, knowledge, experiences, information, and understanding. Education should be tailored to the patient’s knowledge by asking questions about their education level, age, medical history, previous knowledge, and information. Also, they should consider the patient’s learning ability when adjusting the education schedule.

### Strengths and limitations

A significant strength of this study is its novelty. It is the first qualitative study to explore PE experiences among CVD patients based on the andragogy model. In this study, one of the limitations was the need to spend adequate time with patients to conduct interviews thoroughly and to identify patients who have had a sufficient and comprehensive experience with PE. It is also important to note that qualitative studies limit the possibility of generalizing findings. However, the results of this research are not intended to be generalized.

## Conclusion

This study shows that the principles of andragogy are not seriously considered in providing PE to adult patients. The primary reasons for such inattention may be education providers’ lack of knowledge about and non-adherence to PE guidelines and models. As most CVD patients are adults, developing and providing PE based on the principles of andragogy can improve the quality of education and facilitate the attainment of health promotional and educational aims.

## Data Availability

The data used to support the findings of this study are available from the corresponding author upon request.

## Abbreviations

CVD: Cardiovascular disease
CVDs: Cardiovascular diseases
WHO: World Health Organization
SBMU: Shahid Beheshti University of Medical Sciences
SPSS: Statistical Package for Social Sciences.
